# The emerging role of neutrophil extracellular traps in cancer: from lab to ward

**DOI:** 10.3389/fonc.2023.1163802

**Published:** 2023-04-28

**Authors:** Wentao Zhong, Qianyu Wang, Xiaofei Shen, Junfeng Du

**Affiliations:** ^1^ The Second School of Clinical Medicine, Southern Medical University, Guangzhou, China; ^2^ The Second School of Clinical Medical, Shanxi Medical University, Taiyuan, China; ^3^ Department of General Surgery, Affiliated Drum Tower Hospital of Nanjing University Medical School, Nanjing, China; ^4^ Medical Department of General Surgery, The 1st Medical Center, Chinese PLA General Hospital, Beijing, China; ^5^ Department of General Surgery, The 7th Medical Center, Chinese PLA General Hospital, Beijing, China

**Keywords:** cancer, therapeutics, tumor microenvironment, neutrophil extracellular traps, metastasis (cancer metastasis)

## Abstract

Neutrophil extracellular traps (NETs) are web-like structures derived from neutrophils, which typically consist of DNA, released from the nucleus or mitochondria, and decorated with histones and granule proteins. They are well known as an important structure in innate immunity to eliminate pathogenic bacteria, similar to neutrophils. Initially, NETs are reported to take part in the progression of inflammatory diseases; now, they have also been implicated in the progression of sterile inflammation such as autoimmune disease, diabetes, and cancer. In this review, we will describe the recent studies which have investigated the role of NETs in the development of cancer, especially metastasis. We also prescribe the strategies for targeting NETs in the multiple cancer types, which suggest that NETs are a promising treatment for cancer patients.

## 1 Introduction

There are many ways to cure cancer, including surgery, chemotherapy, radiotherapy, and immunotherapy, however, recurrence and metastasis are still the main reason for the low survival rate of patients (1). The tumor microenvironment (TME) comprises all the non-cancerous host cells in the tumor, including fibroblasts, endothelial cells, neurons, adipocytes, adaptive, and innate immune cells, as well as its non-cellular components, including the extracellular matrix (ECM), and soluble products such as chemokines, cytokines, growth factors, and extracellular vesicles. The constant interactions between tumor cells and the TME play a decisive role in tumor initiation, progression, metastasis, and response to therapies (2, 3).

Neutrophils which take part in the pathogenesis of numerous diseases are the essential players in the early response against pathogens and during acute inflammation and play an important role in the regulation of innate and adaptive immune responses (4, 5). Recently, cancer-associated inflammation has been recognized as a hallmark of tumor biology (6). An inflammatory response to a tumor will contribute to cancer initiation and progression, allowing tumor cells to escape elimination by the immune system. Recent studies showed that neutrophils are an important component of the TME and have highlighted their importance in tumor progression and therapy (7–9). Due to the heterogeneity and plasticity of neutrophils, when receiving different external incentives, tumor-associated neutrophils (TAN) are polarized into antitumor and pro-tumor populations, which are named TAN-N1 and TAN-N2 (10). In 2004, Brinkmann et al. (11) first described that neutrophil extracellular traps (NETs), extracellular fibers released from neutrophils, consist of granule proteins and chromatin, bind Gram-positive and -negative bacteria, and are vital components of the innate response. The pathway of NET production has been described as a new form of cell death, NETosis, distinct from apoptosis and necrosis (12). In addition to their important role in defense capability, NETs also play an important role in the TME (13). In this review, we will not only introduce how the NETs are produced by neutrophils but describe the crosstalk between NETs and tumor cells and the prognostic significance of NETs on cancer patients intensively.

## 2 An overview of nets

Previous research on neutrophils and their product, NETs, have mainly focused on inflammatory diseases, including sepsis and wound. In recent years, as studies involving neutrophils have intensified, it has been discovered that neutrophils and NETs are implicated in the progression of sterile inflammation including autoimmune disease, diabetes, and cancer (14–16).

NETs are unique net-like structure in the organism that originated from neutrophils. Like neutrophils, they act as the first defense of the organism against external stress, playing a part in removing foreign pathogens. The progression of NET formation was first described in 2004 (11); neutrophils are activated by external factors such as lipopolysaccharide (LPS) and phorbol myristate acetate (PMA) and then release intracellular DNA, histones, and granule proteins such as myeloperoxidase (MPO) and neutrophil elastase (NE) (17). These substances constitute the NETs in the extracellular compartment, and this special structure can be observed under the electron microscope. They play an important role in regulating the biological behavior of the tumor, especially tumor metastasis (18, 19).

**Figure 1:**
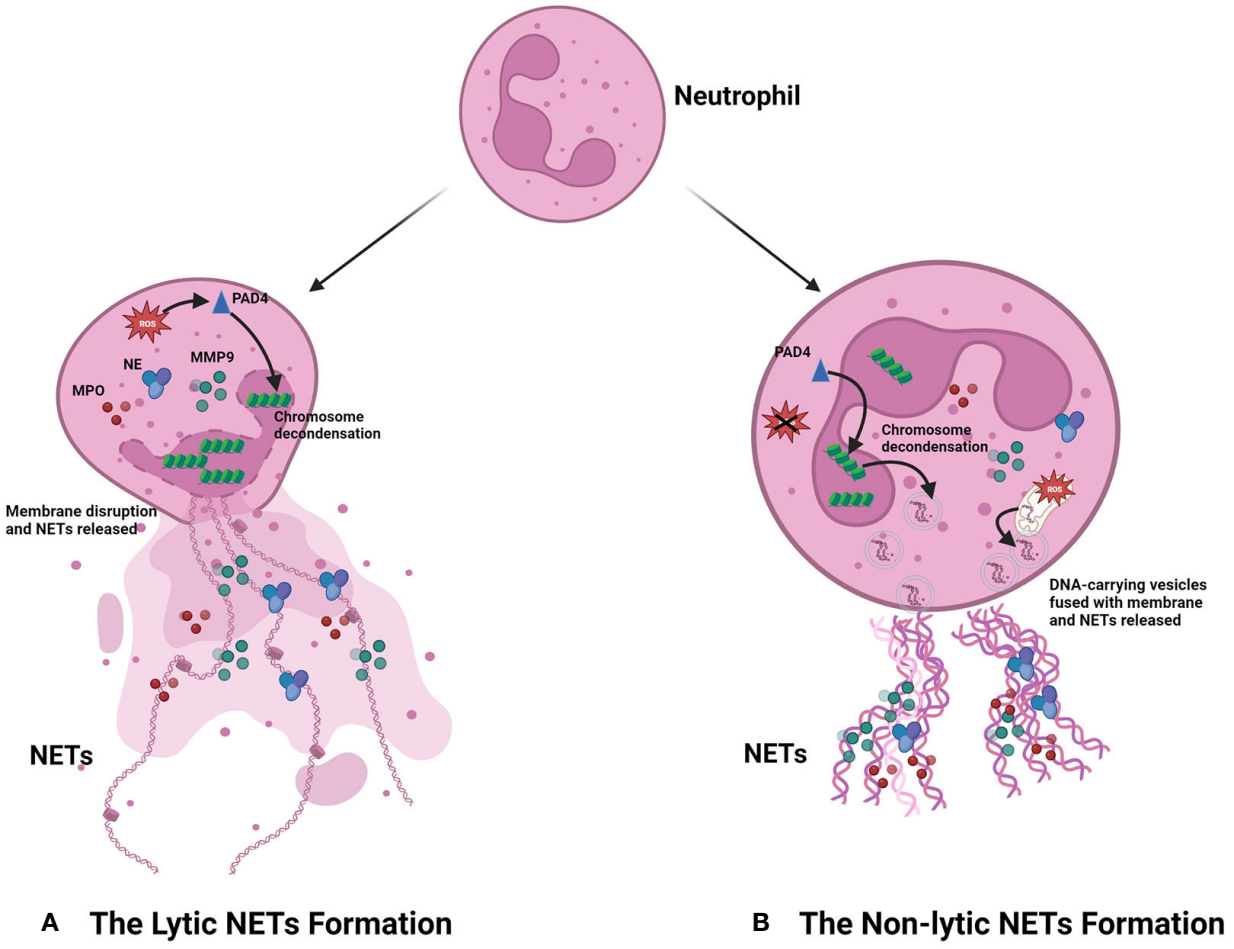
Progression of NET formation. When neutrophils are stimulated by stimulus, they can produce NETs in two main ways according to the different destinies. **(A)** The lytic NET formation. When stimuli including LPS, PMA, and IL-8 bind to the receptors of neutrophils, NADPH-oxidase is active, which can increase the level of ROS. Then, the increased ROS promotes chromosome decondensation by activating PAD4. The DNA derived from the chromosome is decorated with granule proteins and forms NETs, which are released from the dead neutrophil. **(B)** The non-lytic NET formation. The stimulus including damage-associated molecular patterns (DAMPs), bacteria, and injury can promote NET formation in neutrophils by forming vesicles. Different from the destiny of neutrophils in the lytic NET formation, these neutrophils still preserve intact membranes and the phagocytic function. Notably, the DNA in NETs derived from the chromosome of the nucleus is ROS-independent; however, the formation of NETs comprising mitochondrial DNA is ROS-dependent.

The process of NET formation is known as “NETosis,” and there are two forms of NETosis based on whether the neutrophils lyse and die after the generation of NETs (**Figure 1**). The first is lytic NETosis, in which the neutrophil plasma membrane is cleaved and dies after the formation of NETs, and it lasts around 2–4 h. The other form is non-lytic NETosis, a new way different from lytic NETosis, described by Yipp et al. (20). During the pathway, neutrophils do not lyse and die after generating NETs; instead, they preserve the phagocytic function of normal neutrophils, and it starts within 1 h after stimulated by *Staphylococcus aureus* (21) and *Candida albicans* (22).

As the most extensively studied procedure for the formation of NETs, the stimuli of the lytic NETosis mainly include PMA, LPS, and interleukin-8 (IL-8), which, upon contact with neutrophils, initiate intracellular generation of reactive oxygen species (ROS) in an NADPH-dependent way, followed by activation of the peptidyl arginine deiminase 4 (PAD4), an essential enzyme in the NETosis process. PAD4 can facilitate chromatin decondensation, which allows DNA and histones to be excreted outside the cell and constitute the framework of NETs. It also activates a diverse range of granule proteins in neutrophils, such as NE and MPO, which bind to DNA extracellularly and collectively form the NETs (15). However, different from lytic NETosis, when external stimuli provoke non-lytic NETosis, neutrophils can form DNA-carrying vesicles independent of ROS. These vesicles can then merge with the cytosolic membrane and deliver DNA to the extracellular space, after which it is combined with granule proteins from the neutrophils to form NETs (23, 24). In addition, the researchers have also discovered that the DNA within NETs can come to be originated not only from the nucleus but also from the mitochondria, an organelle containing low amounts of DNA (25–27). Between the two pathways, the most studied is the lytic NET formation, which is also mainly discussed in this review.

## 3 How nets promote tumor growth and metastasis

### 3.1 Crosstalk between tumor cells and NETs

**Figure 2:**
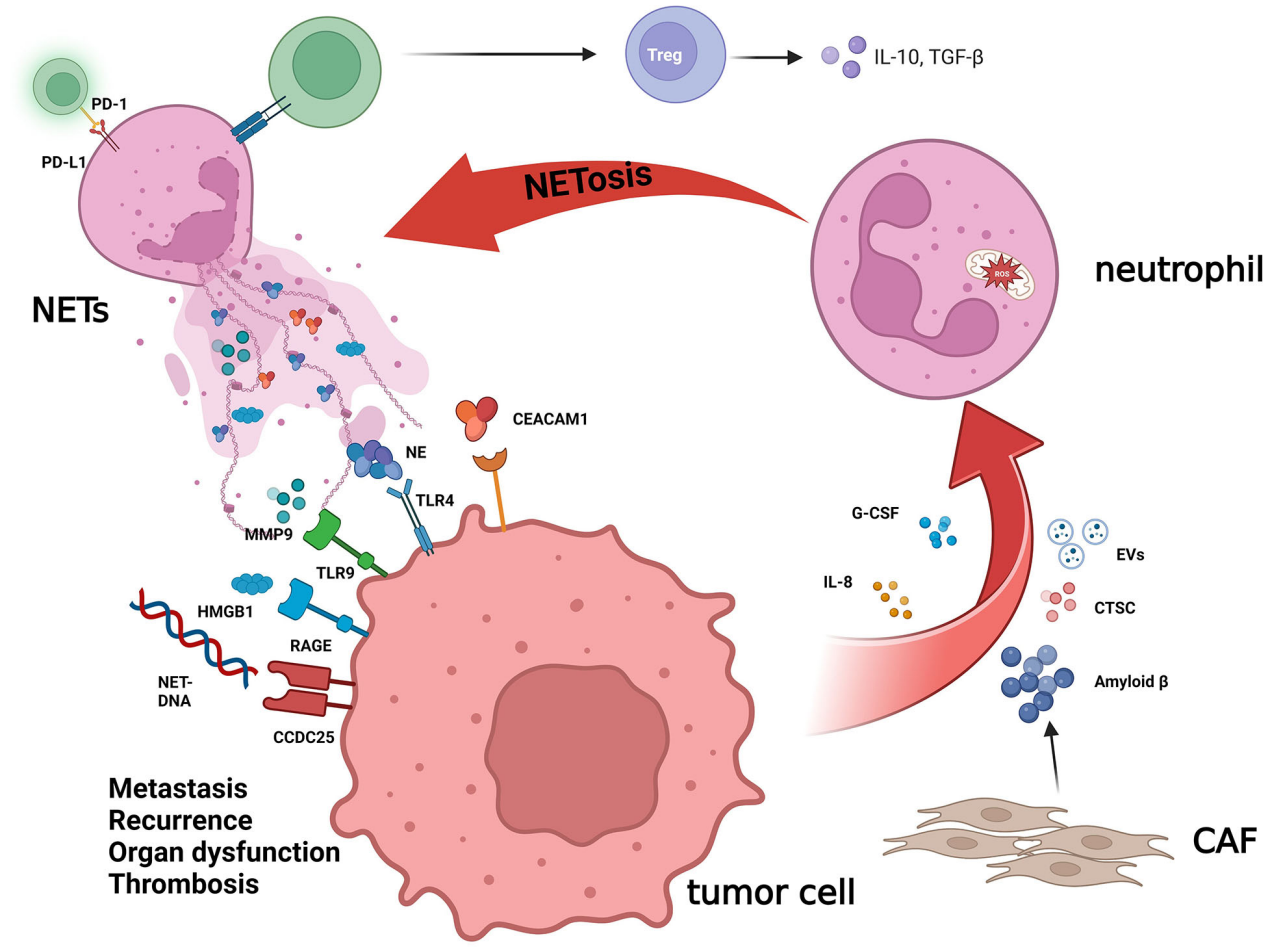
The crucial role of NETs in cancer biology. NETs, a net-like structure produced by neutrophils, play an important role in TME, which can influence cancer biology by cross-talking with tumor cells. NET-DNA, which is the main component of NETs, can interact with CCDC25 on the cytomembrane of tumor cells and then activate the ILK-β-Parvin pathway to promote cell motility. NETs are decorated with HMGB1, and RAGE is the major receptor for HMGB1 in mediating sterile inflammation. The NE, MMP9, and CEACAM1 released by NETs trigger the TLR-4 and TLR-9 receptors on cancer cells, accelerating the growth, metastasis, and recurrence of the tumor by altering the metabolism and “waking up” dormant tumor cells. Other components in the TME also have a mutual effect with NETs. The amyloid β derived from CAF and other factors produced by tumor cells, including IL-8, G-CSF, CTSC, and EVs, will increase the level of NETs. NETs can also promote the differentiation of Treg cells and affect the immune-modulating function of T cells.

The interaction between tumor cells and NETs includes several different ways (**Figure 2**). First, NETs can activate a variety of receptors and signal pathways associated with growth, and metastasis to shape the characteristics of the tumor. High mobility group box 1 (HMGB1), a protein widely distributed in the body, has been discovered to have the pro-inflammatory function and becomes in recent years one of the popular targets of research in critical care medicine, and NETs also seem to enhance the malignancy of cancer (28), for the possible reason, just like Zhang et al. reported, that HMGB1 activates the nuclear factor-kappa B (NF-κB) signaling pathway upon binding to the receptor for advanced glycation end products (RAGE) on the tumor cell surface and promotes tumor secretion of IL-8 (29, 30). In contrast, IL-8 recruits neutrophils and promotes the production of NETs, thereby creating a positive feedback, which also promotes colorectal cancer liver metastasis (31). The study by Tohme et al. (32) has indicated that NETs can promote HMGB1 production within tumor cells and activate TLR9-dependent pathways to promote tumor cell growth, metastasis, and invasive ability. Furthermore, the binding of NETs to tumor cells can also induce tumor cells to acquire resistance to death as well as enhanced invasiveness by activating the TLR4/9-COX2 pathway, and the use of DNase I in combination with the anti-inflammatory drugs can effectively reduce hepatocellular carcinoma metastasis (33). According to Albrengues et al. (34), NETs can also “wake up” dormant tumor cells through metalloproteinase (MMP) and NE, facilitating metastasis and recurrence.

Similarly, tumor cells can also impact the formation of NETs by secreting some cytokines and proteins, the most investigated of which are IL-8 and granulocyte colony-stimulating factor (G-CSF) (30, 35–37). Xiao et al. (38) reported that cathepsin C (CTSC), the protease produced by tumor cells, can activate proteinase 3 (PR3) on the neutrophil membrane, to promote interleukin-1β (IL-1β) to process and NF-κB to activate, which can upregulate interleukin-6 (IL-6) and CCL3, recruit neutrophils, and promote the production of ROS in neutrophils to induce NET formation. The extracellular vesicles (EVs) derived from the tumor are deemed to associate with the growth of cancer and modulate the TME and immune function (39, 40). The construction of a mouse model of breast cancer using 4T1 breast cancer cells reveals that 4T1-derived EVs promote the emergence of NETs and accelerate cancer-associated thrombosis in veins (41). Moreover, EVs derived from KRAS-mutated colorectal tumor cells can induce neutrophil recruitment and promote NET formation through IL-8 activation (42). Recently, Guimarães-Bastos et al. (43) revealed that EVs derived from melanoma cells can induce neutrophil chemotaxis, promote TAN polarization to TAN-N2, a pro-tumor population, and facilitate NET formation, thus contributing to tumor progression.

### 3.2 NETs chat with other components

In addition, as part of the tumor microenvironment, NETs can also interact with other tumor components. Compared with the normal, Zhang et al. (44) revealed that Th17 and interleukin-17 (IL-17) levels were significantly increased in pancreatic cancer and further found that the elevation of IL-17 induced neutrophil recruitment and NET production, which in turn had a suppressive effect on CD8+ T cells. Similarly, Kaltenmeier et al. (45) observed that the immune function of T cells is significantly suppressed function in the TME with a high density of NETs. *In vitro*, they found that NETs also contained programmed cell death-ligand 1 (PD-L1), which inhibited T-cell function by combining with programmed cell death protein 1 (PD-1) on the T-cell surface, resulting in T-cell dysfunction and metabolic failure, and hence promoting tumor growth.

Cancer-associated fibroblasts (CAFs) are a common composition within the stroma, affecting tumor angiogenesis, stromal remodeling, and antitumor immunity and promoting tumor invasion (46–48). A recent study revealed the impact of CAFs on the formation of NETs (49); the amyloid-β produced by CAFs is involved in the induction of NETs by tumor cells through promoting intra-neutrophil ROS production and supports cancer progression, whereas NETs can also promote liver metastasis of pancreatic tumors by enhancing the migration ability of hepatic stellate cells and forming CAFs (50). DNA derived from NETs (NET-DNA) can also activate the stellate cells in the pancreas then forming fibrous stroma, promoting and enabling tumor proliferation by activating RAGE (51). CCDC25, a transmembrane protein, is a DNA receptor (52–54) and enhances cell motility by activating the ILK-β-Parvin pathway after binding to NET-DNA. Notably, they suggested that metastasis to the liver, but not other organs, was related to a higher level of NET-DNA in breast and colon cancer patients, implying that tumor metastases could be predicted by detecting NET-DNA content in the blood. In addition, Rayes et al. (55) prevented the metastasis of colon cancer by blocking carcinoembryonic Ag cell adhesion molecule 1 (CEACAM1), a component protein of NETs.

### 3.3 NETs promote metabolic reprogramming

Tumor cells can escape the immune clearance of the body through metabolic reprogramming. Variations in tumor metabolic pathways, including those favoring mitochondrial metabolism as well as oxidative phosphorylation, may allow tumor cells in the TME to cope better with stress (56). New studies show that NETs have an effect on the metabolism of tumor cells (57). NE in NETs can activate TLR-4 on the surface of tumor cells, leading to an accumulation of intracellular PGC1a levels, which enhances the function of mitochondria and accelerates the growth of cancer.

In addition to directly affecting tumor cells’ metabolism, NETs can also change the metabolism of immune cells. Non-alcoholic steatohepatitis (NASH) can be developed into hepatocellular carcinoma (HCC) with or without cirrhosis, Tsung et al. (58) found that those mice whose non-alcoholic steatohepatitis (NASH) was induced by a high-fat diet had greater NETs in the liver at an early stage, which could recruit the macrophages and promote the evolution of liver cancer. Inhibiting NET formation would not influence the development of a fatty liver but decrease the evolution of HCC. Subsequently, Wang et al. (59) used the FoxP3-DTR mouse model to simulate Treg cell clearance and discovered that NETs could induce the development of NASH to HCC by promoting the oxidative phosphorylation of mitochondria within naïve CD4+ T cells and promoting their conversion to Treg cells, which also indicated that NETs could promote the connection between the innate and adaptive immune. Zenlander et al. (60), however, found no significant difference in the levels of NETs in patients with liver cancer that developed from cirrhosis compared with those with only cirrhosis. Also, tumor cells can promote NET production in a ROS-dependent pathway by inducing a transition from TAN to glycolytic and pentose phosphate metabolic pathways (61).

## 4 Clinical significance of nets

### 4.1 NETs in the tumor microenvironment

Recently, there has been growing research suggesting that NETs are important part of the tumor microenvironment after discharging from neutrophils and can influence not only the progression of the tumor but also the metastasis and therapy, especially the metastasis of cancer.

It was found that, compared with the normal tissues, the density of NETs was significantly higher in patients with breast, gastric, and lung cancers (62–64). Yang et al. (52) also found that NETs were abundant within liver metastases in cancer patients and the NET levels could be used for early prediction of liver metastases in breast cancer patients. Similarly, it has been reported that the peripheral blood neutrophil-to-lymphocyte ratio (NLR) correlates obviously with NETs in peripheral blood and the density of NETs tends to be higher in patients with lymph node metastases (65). Remarkably, the distribution of NETs was inconsistent in the tumor and its adjacent paraneoplastic tissues, with the highest density of NETs in the center of the tumor and the tendency for both the density of NETs and neutrophils to decrease from the center of the tumor to the stroma (65). It may be since neutrophils within the tumor are more likely to develop NETs (66); this suggests that intra-tumor NETs may be more capable of influencing the tumor. Nevertheless, it was found that cervical cancer with a high density of NETs in the stroma had a better prognosis. At the same time, the level of NET intratumor did not affect the patient’s prognosis (67). Surendran et al. (68) developed a new three-dimensional (3D) tumor-immune microenvironment (TIME)-on-Chip device, which can simulate the TME *in vitro* to observe the neutrophil response during tumor cell proliferation and invasion. As a result, it was observed that NETs were formed when neutrophils came into contact with tumor cells and that NETs promoted tumor cell clustering and invasion into the stroma, which was more evident with NETs in the stroma. Therefore, it would be a promising field to explore the prognostic effects of NETs at different locations of the tumor.

Tumor metastasis is often the cause of poor prognosis for patients, including lymphatic metastasis and distant organ metastasis; the recurrence and metastasis of tumors can also be associated with NETs. First, epithelial–mesenchymal transition (EMT), a common cause of metastasis, has been demonstrated to be modulated by NETs. Metastasis is always under the regulation of TME changes like inflammation, intravasation of angiogenesis, and cancerous cells, which is described as EMT. EMT enables epithelial cells to obtain a mesenchymal cell phenotype, accelerating the entry of tumor cells into the vascular system and leading to distant metastases (69, 70). Using purified NETs cocultured with colorectal cancer cell lines, Stehr et al. found that NETs could promote the cell motility of CRC cells (71), which was correlated with more mesenchymal biomarkers, and EMT increased the transcription factors while reducing the level of the epithelial biomarkers, such as E-cadherin (CDH1) and epithelial cell adhesion molecule (EPCAM). Similarly, the same results were observed in gastric cancer (63), pancreatic cancer (28), and lung cancer (72). NETs can promote EMT and metastasis in non-small cell lung cancer by inhibiting long non-coding RNA (lncRNA) MIR503HG expression and activating the NF-κB pathway. NETs have also been shown to promote tumor cell entry into the circulatory system by downregulating intercellular tight-junction molecules (73–75). As an important component of the innate immune system, the complement system is significant in the process of tumor growth. Liu et al. (76) found that complement factor 5a (C5a), the downstream product of the C3b-catalyzed cleavage of C5, can recruit neutrophils, and membrane attack complex (MAC), a multiprotein containing several complement compositions, can promote NET formation by activating neutrophils that have contact with vascular endothelium. Then, NETs destroy the endothelial barrier and enhance vascular leakage, facilitating the entry of tumor cells into the blood and causing distant metastasis. Depleting the neutrophils or inhibiting the formation of MAC gives protection to the vascular endothelium and prevents the metastasis.

The alteration of the microenvironment in distant metastatic organs before tumor metastasis is known as pre-metastatic niche (PMN), and it helps to attract circulating tumor cells (CTCs) (77), thus promoting metastasis. There are six characteristics of PMN, namely, immunosuppression, angiogenesis/vascular permeability, inflammation, lymphangiogenesis, organotropism, and metabolic reprogramming. Zeng et al. (78) demonstrated that *in situ* breast cancer cells can enhance the level of hydroxy acid oxidase 1 (HAO1) in the lung, the rate-limiting enzyme of oxalate metabolism, and promote oxalate generation by activating the TLR3-IRF3 signaling pathway. Oxalate not only promotes the growth of metastatic tumor cells through the MAPK pathway but also activates NADPH oxidase, leading to increased ROS production, thus inducing the production of NETs and promoting the formation of PMN in the lung, making breast cancer more prone to metastasis to the lung. In addition, mesenchymal stem cells (MSCs) in the lung have potent pro-metastatic properties (79). Th2 cells in the lung induce C3 synthesis by MSCs through STAT6, which can induce neutrophil recruitment and NET formation to promote metastasis. By blocking the Th2-STAT6-C3-NET pathway, lung metastasis driven by MSCs was also attenuated. A particular type of neutrophil population was recently identified (80), tumor-associated aged neutrophils, whose cell marker is CXCR4hiCD62Llo. In a constructed tumor metastasis model, tumor cells lead to the accumulation of aged neutrophils by disrupting neutrophil homeostasis and directly stimulating neutrophil aging regulated by angiotensin II. The aged neutrophils can release multiple metastasis-promoting factors like NETs and MMP9, and aged neutrophil permutation can significantly increase liver metastasis of breast cancer and melanoma, which are mediated mainly by NETs. It has been observed that these cells are present not only early in the pre-metastatic microenvironment of the lung but also in the peripheral blood of patients (81). Aged neutrophils induce mitochondrial DNA release by sirtuin 1 (SIRT1), thereby inducing the formation of NETs, rather than the traditional Cit-Histone H3-dependent lytic NET formation, promoting breast cancer lung metastasis. Earlier research also found that the presence of NETs in the peritoneum as well as in the omentum contributes to PMN formation and promotes tumor metastasis (82, 83).

In addition to the above factors contributing to tumor metastasis, surgical operation and postoperative infection have been reported as risk factors for recurrence and metastasis in postoperative patients. For the majority of solid tumors, surgery is the preferred treatment to improve the prognosis of patients (84). However, the study found that whereas the operation removes the primary tumor, it is also deemed to promote the eruption of undetected microscopic lesions, increasing the possibility of recurrence and metastasis after surgery (85), and NETs also participated in the process (86). During the operation, with the destruction of the tumor and its associated blood vessels, some of the tumor cells can flow into the circulation system to form the CTCs, which act as “seeds” in the process of cancer recurrence and metastasis (87). In addition, tissue damage caused by surgery activates the immune and coagulation systems of the body, in which neutrophils, NETs (88), and platelets (89–91) can promote the tissue healing process, but they may also contribute to the spreading and metastasis of tumor. When CTCs enter the peripheral blood, they are rapidly coated by platelets to protect them from external stress and destruction by NK cells. Ren et al. (89) simulated the effects of surgery on the organism by constructing a model of liver ischemia–reperfusion injury (I/R) and showed that platelets were activated by local inflammation caused in I/R through the TLR4-ERK5 pathway and then bound to CTCs to form platelet-tumor cell clusters and that integrins could facilitate the connection of clusters and NETs and promote metastasis (92). In addition to the inflammatory response to tissue damage caused by surgery, postoperative infection, one of the common complications of the surgery, also promotes tumor recurrence and metastasis to some extent (93, 94). Postoperative peritoneal infection in gastric cancer induces NET formation and promotes gastric cancer invasion and metastasis by activating the TGF-β signaling pathway (95). Wang et al. have certificated that elevated LPS levels caused by a postoperative infection in colorectal cancer can induce NET production through the activation of TLR-9 and MAPK signaling pathways, which are closely associated with increased postoperative recurrence rates (96).

The link between cancer and thrombosis has been discovered for decades (97, 98), whereas increased levels of blood clotting factors, tissue factor, and activation of fibrous protein has been described as the mechanism. However, the exact mechanism causing this change is not known. Fuchs et al. (99) revealed that NETs can promote platelet adhesion, aggregation, and activation in the vasculature and induce thrombosis. In the research of Demers et al. (100) in 2012, it was first described that induction of NETs by G-CSF, which was derived from tumor cells, could promote coagulation in tumor patients, leading to cancer-associated thrombosis formation. Subsequent studies (101–104) also demonstrated that neutrophils and NETs contribute to platelet activation and tissue factor synthesis, leading to the formation of venous thrombosis in cancer patients. However, it has been shown in other investigations that NETs only affect the formation of atherothrombosis in tumor patients and do not affect venous thrombosis (41, 105). Therefore, whether NETs affect venous thrombosis in cancer patients and the specific mechanisms involved need further investigation. Other than promoting the formation of cancer-associated thrombosis, some researchers have recently suggested that NETs may also affect the myocardium (106). Using mice with breast cancer, they found a correlation between myocardial dysfunction and NETs in mice, and inhibiting the formation of NETs improved the inflammatory response of the myocardium and decreased the level of biomarkers.

### 4.2 NETs in cancer therapy

NETs have been previously investigated in sepsis and other inflammatory diseases and can be a more promising target for cancer therapy based on their proliferation-promoting and metastatic effects on tumors as well as their impact on the TME. The current therapeutic approach to NETs consists of two main aspects, inhibition of NET formation or destruction of formed NETs. NET-DNA from the nucleus and mitochondria, which is the vital composition of NETs, can be hydrolyzed by DNase I. NET-DNA can accelerate the growth and metastasis of cancer after binding to CCDC25, and destroying NETs by using DNase I is frequently used in current trials (107, 108). Many recent studies proved that heparin can promote the degradation of NETs by detaching histones from the NET-DNA skeleton (99); the use of low molecular heparin can hinder the formation of NETs induced by PMA (109). However, a recent study found that heparin can induce NET formation *in vitro* (110). This suggests that further research is expected to confirm whether low molecular heparin can be used to degrade NETs and by what mechanism.

Interfering with the formation of NETs can be a positive strategy instead of degrading the formed NETs by suppressing the compositions crucial for the NETs, such as PAD4, NE, or MPO (**Table 1**). Chromatin densification is the most pivotal process in the formation of NETs, and it is dependent on the existence of PAD4 (57). Lewis et al. (114) have recommended two inhibitors of the PAD4, especially GSK484, which can suppress disease by destroying NETs. NE and MPO are the critical compositions of NETs, and mouse models lacking NE have been utilized to study the effects of decreased NETs on cancer metastasis (18, 86) and sepsis (123). With regard to MPO, which is usually viewed as a marker of NETs, it can also influence the NETs. Mice treated with MPO inhibitors could not form NETs and are always utilized to observe the impacts of NETs on cancer. Based on the increasing research on NETs and tumors, specific blockade of the interplay has become a new therapeutic option. Blocking NETs or tumor cell-derived factors, including IL-8, IL-17, and their receptors, has been shown to affect the biological behavior of cancer (44, 124–126).

**Table 1:** Targeting NETs in multiple cancer types.

Major impact	Targets	Inhibitors	Cancer type	Reference
Inhibiting NET formation	NADPH	Kaempferol	Breast cancer	(111)
	PAD4	BMS-P5	Multiple myeloma	(112)
		CI-amidine	Chronic myeloid leukemia	(113)
		GSK484	Cancer-associated kidney injury	(114, 115)
		JBI-589	Lung and colon cancer	(116)
Accelerating NET destruction	NET-DNA	DNase	Breast cancer; colorectal cancer; hepatocellular carcinoma; cancer-associated thrombosis	(73, 107, 117, 118)
	NE	GSDMD	Melanoma	(119)
		Sivelestat	Lung and colon cancer; gastric cancer	(18, 120)
		GW311616A	Large B-cell lymphoma	(121)
Blocking the pathway	NF-κB	NBD peptide	Breast cancer	(30)
	TLR-9	Hydroxychloroquine	HCC; pancreatic cancer	(33, 102)
	DDR1	7rh benzamide	Pancreatic cancer	(122)

Furthermore, NETs have been demonstrated to play a role in tumor treatment resistance, including chemotherapy resistance (127), immunotherapy resistance (44, 128, 129), and radiation therapy resistance (130). NETs were observed in radiation-resistant bladder cancer patients compared with the radiation-sensitive patients, and inhibiting HMGB1 and NETs significantly improved the outcome of radiation therapy (130). The TME has a vital role in cancer immunity and probably helps to inhibit the effects of immune checkpoint inhibitors and other new immunotherapies in terminal cancer patients. Zhang et al. (129) used DNase I to degrades NETs that could decrease the resistance to anti-PD-1 therapy in a CRC model. However, a study by Liu et al. (131) indicated that NETs show a novel immunomodulatory role in Bacillus Calmette-Guerin (BCG) immunotherapy. Tumor cells activated by BCG can induce NETs through their production of IL-8 and TNFα, and these NETs help to recruit T cells and macrophages and repair damaged tissue, inducing tumor cell apoptosis and cell cycle arrest.

## 5 Conclusion

Despite the multiple treatment options available for cancer, recurrence, and metastasis are currently still the most common cause of patient death. With intensive research in recent years, the TME has been recognized as the main influencing factor of tumor behavior. The cellular debris produced by apoptosis and necrosis of tumor cells leads to local inflammatory reactions, activating the innate immune response and recruiting neutrophils. NETosis is a vital way by which neutrophils function, but research on the impacts of NETs on tumor cells is still in its infancy. Although a few studies have suggested that NETs have some antitumor effects, it is clear that NETs play a role in promoting the proliferation, invasion, and metastasis of tumors, and even the effectiveness of chemotherapy, radiotherapy, and immunotherapy on cancer patients is related to NETs.

There are mounts of mechanisms underlying NET-dependent tumor progression and metastasis. As reviewed herein, NETs and tumor cells in the TME have been shown to interact through the production of multiple factors, proteins, and their receptors. All results of different studies listed in this review imply the need for further studies about the interaction between NETs and tumors. Although there are some indications that NETs are associated with prognosis in cancer patients, there is still a lack of relevant clinical trials. In addition, it should be noted that neutrophils and NETs are important components of innate immunity, and inhibition of NET formation or destruction of formed NETs may affect neutrophils and reduce pathogenic clearance. Hence, the development of therapies that can accurately target NETs within the tumor without adversely affecting immune function is necessary. Taken together, the emerging role of NETs in cancer diagnosis, growth, invasion, metastasis, and therapy should attract enough attention.

## Author contributions

WZ designed and wrote the manuscript and drafted the figures, QW searched relevant literature. XS and JD supervised this review. All authors have read and agreed to the published version of the manuscript.
